# The magnetic field strength and the force distance dependency of the magnetically controlled growing rods used for early onset scoliosis

**DOI:** 10.1038/s41598-023-30232-8

**Published:** 2023-02-21

**Authors:** Lars Diekhöner, Charlotte Sommer Meyer, Søren Eiskjær

**Affiliations:** 1 Physics Group, Department of Materials and Production, Aalborg University, Skjernvej 4A, 9220 Aalborg Ø, Denmark; 2 Department of Orthopedic Surgery, Aalborg University Hospital, Hobrovej 18-22, 9000 Aalborg C, Denmark; 3 Department of Clinical Medicine, Faculty of Medicine, Søndre Skovvej 15, 9000 Aalborg C, Denmark; 4 Aalborg University Hospital, Hobrovej 18-22, 9000 Aalborg C, Denmark

**Keywords:** Medical research, Physics

## Abstract

Magnetically controlled growing rods (MCGR’s) have revolutionized the treatment of early-onset scoliosis (EOS) because painless lengthenings can be done in the outpatient clinic without anesthesia. Untreated EOS leads to respiratory insufficiency and reduced life expectancy. However, MCGR’s have inherent complications like non-functioning of the lengthening mechanism. We quantify an important failure mechanism and give advice on how to avoid this complication. The magnetic field strength was measured on new/explanted rods at different distances between the external remote controller and the MCGR and likewise in patients before/after distractions. The magnetic field strength of the internal actuator decayed fast with increasing distances and plateaued at 25–30 mm approximating zero. Two new and 12 explanted MCGRs was used for the lab measurements of the elicited force using a forcemeter. At a distance of 25 mm, the force was reduced to approximately 40% (ca. 100 N) compared to zero distance (ca. 250 N), most so for explanted rods. This is used to point out the importance of minimizing the implantation depth to ensure proper functionality of the rod lengthening in clinical use for EOS patients. A distance of 25 mm from skin to MCGR should be considered a relative contraindication to clinical use in EOS patients.

## Introduction

The treatment of early onset scoliosis (EOS) by magnetically controlled growing rods (MCGR) has become the standard of care since the first article by Cheung et al.^1^. Early-onset scoliosis (EOS), defined as scoliosis (Cobb angle/curvature in the frontal plane > 10°) that presents itself before the age of 10^2^, is a rare, but often severe condition. It is well-known that left untreated, EOS may lead to increased disability and potentially life-threatening conditions such as respiratory insufficiency and pulmonary hypertension and a reduced life expectancy^3^. The etiologies of EOS are idiopathic, neuromuscular, congenital, and syndromic with a wide spectrum of underlying diagnoses^2^. The treatment goals are three-fold 1) preservation of thoracic growth and lung growth and pulmonary function, 2) preservation of quality of life and 3) correction of deformity^4^.

The main advantage of MCGRs compared to traditional growing rods (TGR) is pain-free lengthening performed in the outpatient clinic every few months after the initial surgery with the help of an external remote controller (ERC). This compares favourably with the TGR, where manual distraction and open surgery in general anaesthesia must be performed every 6–9 months. This increases the risk of wound infection^5^, and the repeated spine surgeries in children have also been shown to have adverse psychological effects^6^. A large multicentre study comparing MCGR with TGR reported improvements in quality-of-life measures and concluded that the reduced number of surgeries lessens the psychosocial burden^7^. However, an increasing number of MCGR failure mechanisms have been observed^8–11^, and approximately 10% of patients undergo unplanned revisions^12^ most often because of anchor failure, failure of the distraction mechanism^13,14^, fracture of the internal magnet^15^ or a non-functioning MCGR^16^. Titanium wear has raised concerns^17^. The forces elicited by the MCGR depends on the distance between the MCGR and the ERC (implant depth) and has not so far been quantified. The amount of lengthening dependent on implant depth has been described by Seidel et al.^18^ even though he offers no direct explanation for his findings and the R^2^ values for the linear regression model are quite low (0.25–0.36) which means that only a fourth to approximately a third of the variance is explained by the linear regression model presented by Seidel et al.^18^. In fact, surprisingly little is known of the interaction between the internal magnet/actuator and the ERC considering that this interaction is essential for the desired effect—rod lengthening. Recently, the initial optimism described above has been dampened by reports of low survival rates of the MCGR lengthening mechanism at 2 years^19^. On the other hand, one could object that this is dependent on the right indications for surgery.

We aim to specify one correct indication for MCGR use by measuring the lengthening forces at varying distances between the ERC and the MCGR to quantify the role of implantation depth. Furthermore, the magnetic field of the magnet in the MCGR as a function of the distance to the magnet was measured in the laboratory and in vivo to provide insight into the magnetic coupling mechanism that drives the distraction.

## Background

By combining the limited information provided by the manufacturer (Nuvasive Inc.) with the literature^13,14^, it is possible to outline the construction of the MCGR, the lengthening mechanism, and the interaction between ERC and MCGR: An internal dipolar magnet mounted on a screw mechanism is brought into rotation, which leads to an extension of the rod that drives the distraction. The rotation is initiated by an interaction with an external rotating magnetic field generated by the ERC. More specifically, when the magnetic dipole moment $\vec{\mu }$ of the internal magnet is exposed to the external field $\vec{B}$ of the ERC, a torque, given by $\vec{\tau } = \vec{\mu } \times \vec{B}$, is generated. This leads to the rotation of the internal magnet. Because the torque depends on the angle between $\vec{B}$ and $\vec{\mu }$, it is necessary to rotate the field continuously to maintain torque and rotation. Furthermore, the strength of the torque depends strongly on the distance between the ERC and the MCGR, as the fields of the internal magnet and the ERC decay with distance, as very briefly given by the manufacturer’s information shown in Table 1. These are not measured values but based on software simulations. It should also be noted that only two data points in the clinically relevant interval are available, further motivating the current study.

**Table 1:** Manufacturers information on magnetic field strength for MCGR and ERC.

Distance (mm)	4.1	25.4	152.4	304.8	609.6
MCGR Implant magnetic field (Gauss)	1490	105	3.7	0.94	0.24
ERC magnetic field (Gauss)	4340	2260	107	17.7	2.4

The zero point of this distance scale is interpreted to be at the centre of the magnet. When plotting the field vs. distance (z) in Fig. 2a we convert to the scale used in our experiments, where z = 0 is set at the surface of the MCGR.

## Methods

**Figure 1:**
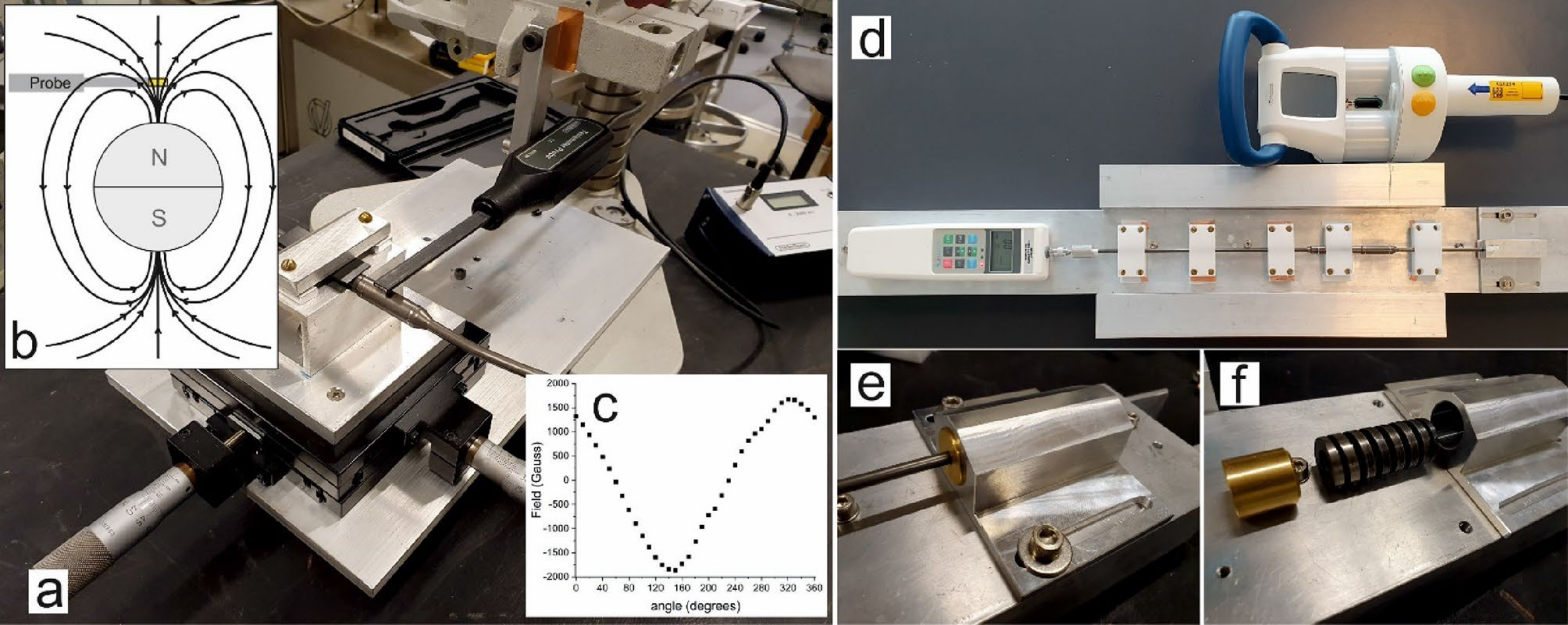
(**a**) Setup for measuring the magnetic field strength as a function of distance. (**b**) Sketch of the magnetic field lines from the dipole in the MCGR and the probe location. (**c**) The measured angular distribution of the magnetic field at the surface of the rod. An angle of 0 degrees corresponds to the orientation shown in b, then the rod is rotated clockwise around its axis. (**d**) Setup for measuring the force generated by the ERC-MCGR interaction. At the left side of the rod, the forcemeter is pictured. (**e**) and (**f**) shows the spring mechanism allowing the rod to extend during interaction.

The a priori hypothesis was that the magnetic field strength of the magnets in MCGRs would decrease with increasing distances between the ERC and the MCGR and likewise that the force elicited by the MCGR would decrease with increasing distances between the ERC and the MCGR. All rods investigated were from the MAGEC® system (Nuvasive Inc., US) with diameters of 4.5 or 5.5 mm (explanted rods) and 4.5 mm (new rods). The diameter at the magnet position was 9.8 mm for all rods. 12 explanted MCGRs from six patients and two new rods were tested using the laboratory setup shown in Fig. 1.

The demographics of the patients and the MCGRs are shown in Table 2. The exact position of the magnet was identified by placing the probe of the magnetometer (Frederiksen Scientific, model 4060.50) above the MCGR and rotating the rod and adjusting the in-plane (x–y) position of the MCGR until the magnetic field strength was maximised. Then, the distance (z) between the magnetometer probe and the MCGR was increased while measuring the magnetic field strength. Zero on the z-scale was defined as the surface of the MCGR. The angular dependence of the magnetic field was measured at the surface (z = 0) of one MCGR by rotating the rod and keeping the probe at a fixed position as shown Fig. 1b,c.

**Table 2:** Demographics.

Patient ID	Etiology	Age in years	Sex	Rod status	Cause of explantation	Rod dimensions	Skin to magnet distance (mm)
1	Cerebral paresis	14	Male	Explanted	Final surgery	5.5 mm/9.8 mm	21.5
2	Myelomeningocele	14	Male	Explanted	Final surgery	5.5 mm/9.8 mm	26.2
3	Cerebral paresis	11	Male	Explanted	Non-functioning	5.5 mm/9.8 mm	16.2
4	SMA	10	Male	Explanted	Non-functioning	4.5 mm/9.8 mm	28.5
5	Idiopathic scoliosis	11	Female	In use	na	5.5 mm/9.8 mm	5.8
6	Congenital scoliosis	9	Male	In use	na	5.5 mm/9.8 mm	9.9
7	Syndromic scoliosis	11	Female	Explanted	Max. length	4.5 mm/9.8 mm	14.5
8	Cerebral paresis	15	Male	Explanted	Final surgery	4.5 mm/9.8 mm	21.5
9	Syndromic scoliosis	11	Female	In use	na	5.5 mm/9.8 mm	12.5

In four cases, magnetic field measurements were performed on patients before and after lengthening for both the right and left rods. The distance between the skin and magnetometer probe was increased by stacking 5 mm plastic spacers to control the distance (up to 25 mm) from the body surface. The additional distance due to the implant depth was measured using an ultrasonic probe (all measurements took place before the rods were explanted). The magnetometer probe was hand-held tangentially to the body surface immediately above the demarcation of the magnet on the skin and the maximum field value was recorded.

Finally, the coupling between the ERC and MCGR was investigated as a function of the distance between ERC and MCGR by measuring the force elicited by the MCGR during lengthening in a laboratory setup, as shown in Fig. 1d–f.

The MCGR was fixed at both ends and plastic guides along the rod were used to prevent rod-bending during ERC activation. The ERC was placed directly above the magnet, and the distance between the ERC and MCGR was precision controlled using acrylic spacers. The ERC was activated, and the maximum obtainable force was measured with a force meter (Sauter FH500) at increasing distances between the ERC and MCGR (zero distance is the surface of the MCGR). The distraction method used was the distraction-to-stall method—when the maximal distraction force (up to approximately 250 N) is obtained the internal actuator automatically stops and a clunk is felt. Two differently termed rods were used (“standard” and “offset”). In addition to a fixed mount, we also added a spring at one end of the rod (Fig. 1f), inspired by Poon et al*.*^20^, allowing the rod to lengthen during interaction with the ERC like in vivo. Force measurements were carried out on 2 new unused rods and 12 explanted rods. Measurements were repeated 6 times for each new rod and 3 times for each used rod.

The influence of the initial rotational magnet position was also investigated for two magnet positions (field pointing in the direction to the ERC and at 90° to the ERC) of the MCGR.

All measurements involving patients were registered in the electronic health records (supplementing the relatively sparse radiological data measurements). The study was approved by the local ethics board of the Head and Ortho Center of the University Hospital. Informed consent from a parent or legal guardian was obtained in all instances. Besides, all experiments were performed in accordance with the 1964 Helsinki declaration and its later amendments.

## Results

**Figure 2:**
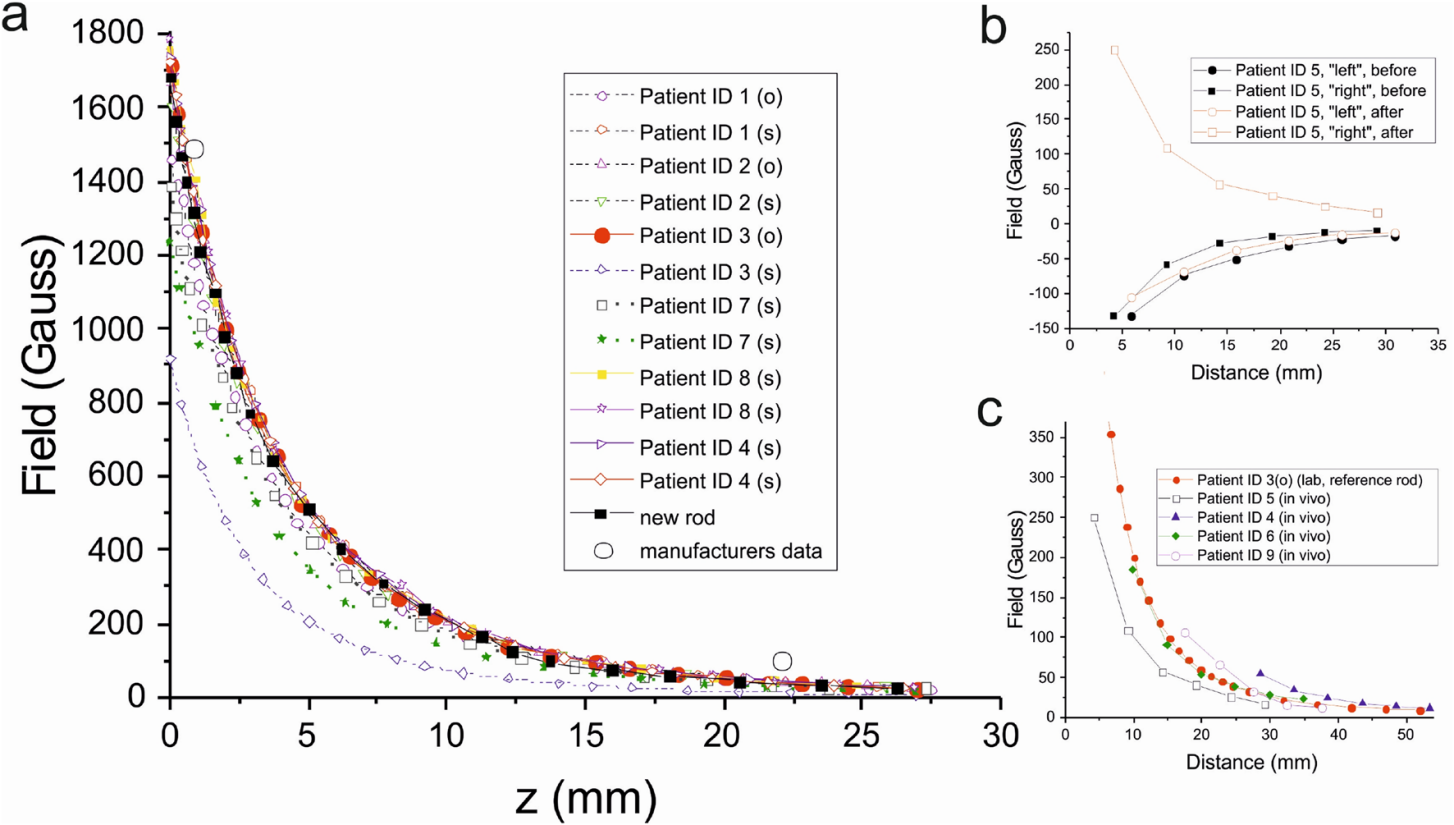
(**a**) Measured field strength as a function of distance for the 12 explanted rods and one new rod. The two datapoints supplied by the manufacturer are also plotted. (**b**) Magnetic field as a function of the total distance to the MCGR surface (in vivo). (**c**) Magnetic field as a function of distance, in vivo and one of the lab measurements for reference. The patient with ID 4 is part of both (**a**)–(**c**) as his rods were explanted after the in vivo measurements.

The magnetic field strength measurements for the 12 explanted rods and one new rod are shown in Fig. 2a. The field decreases with distance, as expected for a dipolar magnet and all data seem to follow the same dependence, except for two rods that had a significantly lower field (ca 30% and 50% reduction). In addition, the two field values available from the manufacturer are also shown.

In Fig. 1c the angular distribution of the magnetic field of the MCGR is shown. We observe an angular distribution as expected for a dipolar magnet, including the change of sign every half turn (180 degrees, see also the sketch of the field lines in Fig. 1b).

Examples of field measurements for randomly selected patients are shown in Fig. 2b,c. A reversion of the magnetic field direction from pre- to post distraction was observed in 3 out of 4 cases, but this just demonstrates that the direction of the magnet is left in a random orientation after lengthening.

In Fig. 2c we compare the measured in vivo field values to one of the explanted rods (reference rod) and obtained very similar measurements (see Supplementary Information, Fig. S1).

**Figure 3:**
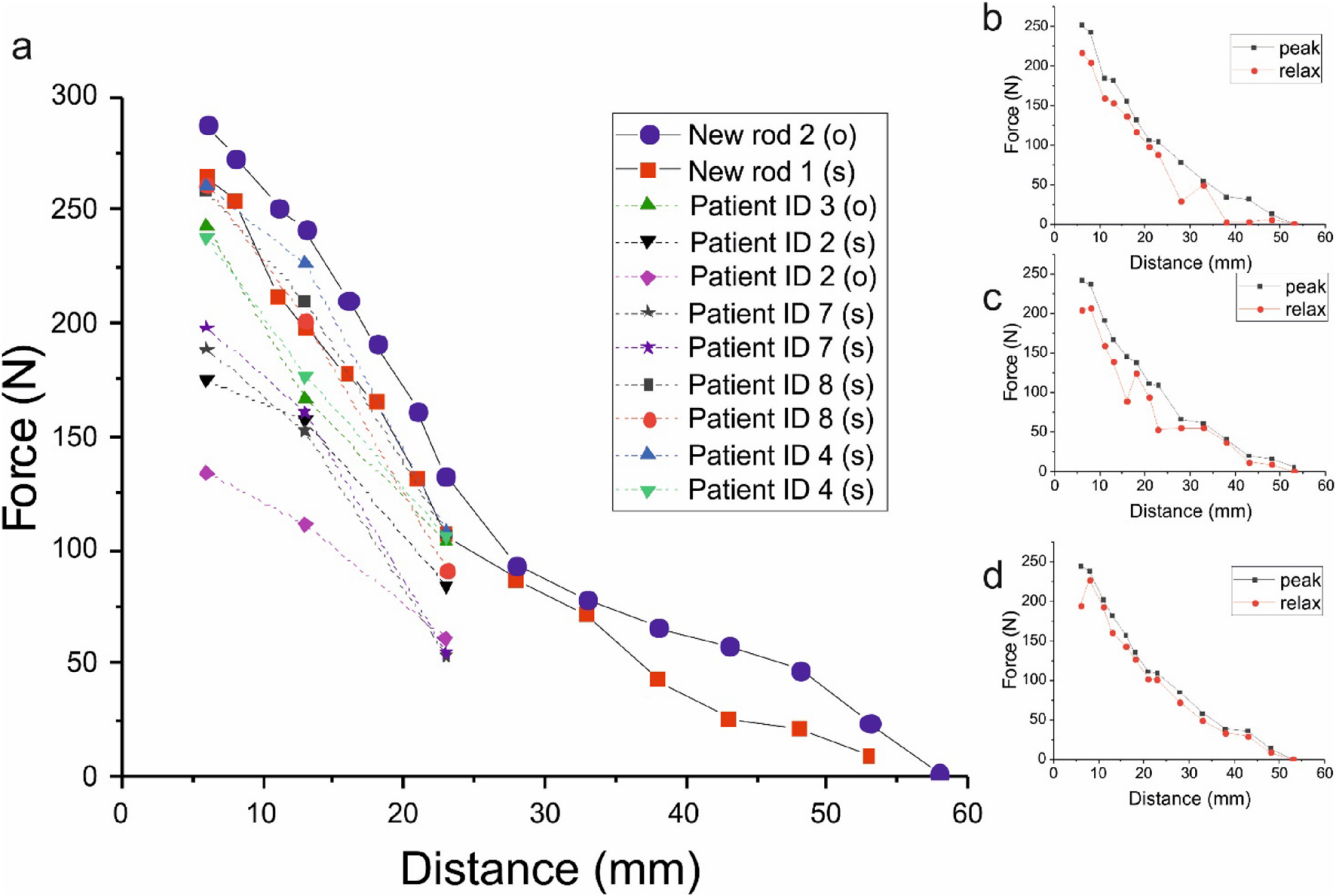
(**a**) Force as a function of distance for two new rods and 9 explanted functioning rods. (**b**)–(**d**) Three consecutive measurements of the peak force during activation and the force value (termed relax) after the ERC is removed. All done on the same new rod (“rod 1”).

The measured forces produced by interaction with the ERC as a function of the distance between the ERC and the MCGR are shown in Fig. 3a. The force values are averaged measurements from six repeated experiments on each of the new rods. For individual measurements see the Supplementary Information (Fig. S2). For the 12 explanted rods (also used for the field measurements shown earlier) it was found that 3 of them produced no force, whereas the remaining 9 produced a force (Table 3; Fig. 3a). Only three distances between the ERC and the rod were measured, and the measurements were repeated three times. Figure 3a demonstrates a clear tendency. For all rods, a very strong distance dependence was observed. Increasing the distance from 6 to 23 mm between the ERC and the MCGR resulted in on average a 57% reduction in the measured force for the new rods and 61% for the explanted functioning rods. Comparing the force values for the 2 new rods and the 9 explanted rods at the 3 distances using the Wilcoxon rank sum test we did not observe a significant difference (confidence interval 95%). Although the p value was 0.14. Figure 3 demonstrates a clear tendency and had we added the 3 zero force measurements the p value would have been significant.

**Table 3:** Force measurements on explanted rods (two rods from each of 6 patients).

Patient ID	Rod type	ERC rod distance 6 mm	ERC rod distance 13 mm	ERC rod distance 23 mm	Time in patient months	Expansion mm
		Force in N	Force in N	Force in N		
1	Standard	0	0	0	37	25
1	Offset	0	0	0	37	22
2	Standard	175	157	84	43	23
2	Offset	134	111	61	43	21
3	Standard	0	0	0	46	23
3	Offset	243	167	104	46	30
4	Standard	260	226	108	49	41
4	Standard	238	177	106	49	34
7	Standard	189	152	53	40	42
7	Standard	198	161	55	40	43
8	Standard	261	202	92	26	24
8	Standard	258	210	107	26	26

Time of use in patients and the total expansion is also given.

All force measurements presented so far are peak values measured during actuation with the ERC. We observed that the force relaxed to a lower value after the actuation was stopped, and the ERC was removed from the rod. Three examples are shown in Fig. 3b–d.

## Discussion

The measurements of the magnetic field strength as a function of the distance to the internal actuator magnet provided detailed insight into the distance dependence of the field (Fig. 2a). There are only two data points available from the software simulations of the manufacturer in the relevant distance range for which good agreement are observed. But it clearly emphasizes the need for the current measurements to map out the full field-distance curve.

The measured magnetic field from the explanted MCGR’s were in accordance with the in vivo measurements from the lengthening sessions (Fig. 2c). Our measurements further show that the fields of the explanted MCGRs are similar to the new rods and confirms that rare-earth magnets do not wear out and function for years beyond the necessary duration of EOS treatment^21^.

A clear failure mechanism we observed was that some of the explanted rods did not produce a force (Table 3), as observed by Rushton^13^. The reason for this was not further investigated because general failure mechanisms have already received much attention^8–11^.

On the new rods we noted that there were considerable variances in the measured forces. The average forces of the two new rods shown in Fig. 3a thus differ substantially at some distances (up to 43 N in absolute numbers), and the six repeated measurements of force vs. distance that the results in Fig. 3a are based on also vary significantly (shown in the Supplementary Information, Fig. S1). The considerable variation in an experimental setup likely increases in vivo. Differences are not due to variances in the field-curves, which are essentially identical (Supplementary, Fig. S2).

It was furthermore investigated if there was a correlation between the measured force for the explanted functioning MCGRs and the time of use in the patients or the total expansion length, but no correlations were observed (see Fig. S3 and Fig S4 in the Supplementary Information).

The current study has shown that the angular position of the magnet is left randomly oriented after each distraction (Fig. 2b). It could be speculated that the varying magnet orientations lead to variances in the coupling between the ERC and MCGR since the magnetic torque generated depends on the angle. We have demonstrated that this is not of importance. Using the force measurement setup shown in Fig. 1d, we deliberately oriented the magnet in different directions and measured the force that could be obtained upon interaction with the ERC. We determined that the forces did not to depend on the initial orientation. Apparently, the ERC works well with all magnet orientations at the typical distances used (10–30 mm were investigated).

We are not aware of other studies quantifying the force distance dependency between the ERC and MCGR, but Seidel et al*.*^18^ have shown that the distance between the ERC and MCGR is one important predictor of obtainable lengthening. Our measurements of force vs. distance clearly demonstrate that the force decreases significantly with distance, and that the distance dependence is quite severe, also at distances typically used in vivo. Thus, the force produced by the MCGR is more than halved at distances around 20–25 mm, where the force produced by the MCGR is approximately 85–120 N. This spells difficulties with increasing number of distractions, as it has been shown in vivo that increasing forces are necessary proportional to the number of lengthenings^22^. However, the interval between lengthenings differs between MCGR’s and TGR’s. Nonetheless, several other studies^23–25^ has corroborated the findings of Noordeen^22^. Mean forces of 200–400 N are required to elongate TGRs 2 mm^22,24^. In several of our patients, we measured distances of 20–30 mm from the skin surface to the MCGR (Table 2) underlining the clinical aspect of the distance problem and this is to some degree corroborated in Table 2 where the functioning rods were the ones with the more modest skin surface to MCGR distances. The laws of physics are valid in MCGR patients as well as the law of diminishing returns^26^. Heyer et al*.* have recently demonstrated that the law of diminishing return is amplified each time the MCGR is replaced^27^. Likewise, a multitude of other factors will work to hinder lengthenings of the MCGRs: hyperkyphosis, high BMI-values, curve stiffness, increase in rod length and curvature, child cooperation etc.^28,29^.

Gaps in the evidence base for the MCGR combined with the frequency of rod malfunctions led to a temporary suspension of the CE certificate in 2021^30^. This study fills a gap in the internal magnet/actuator function which drives the lengthening of the MCGR. This is important as the new European Medical Device Regulation places increased emphasis on the ongoing generation of clinical evidence after certification^31^.

In a consensus study concerning contraindications to MCGR^29^, 71% of respondents agreed that patient size characteristics should be considered a contraindication but could not agree upon a specific BMI range or a specific spinal height range. The current study quantifies the implant depth problem, and our recommendation is that a distance from the skin to the MCGR ≥ 25 mm should be considered a relative contraindication for the use of an MCGR in EOS.

## Conclusions

1. The measured force produced by the MCGR decreased proportionally with increasing distance from the ERC to the MCGR. A 60% reduction in the force elicited by the ERC was seen at a distance of 25 mm. Likewise, the magnetic field strength of the internal magnet/actuator decreased with increasing distances between ERC and MCGR and plateaued at a distance of 25–30 mm approximating zero at this distance.
2. Explanted MCGRs tended to produce lesser force than new unused rods.
3. The polar orientation of the internal magnet/actuator changes randomly after each lengthening but this does not influence the force produced in the MCGR.
4. When the distance between the ERC and the MCGR exceeds 25 mm (skin to MCGR distance) our advice is to carefully consider whether MCGRs is the best choice of treatment.

## Supplementary Information

Supplementary Figures.

## Data Availability

All materials described in the manuscript, including all relevant raw data, will be freely available to any researcher wishing to use them for non-commercial purposes, without breaching participant confidentiality. The corresponding author S.E. should be contacted by anyone wishing to obtain access to the data. On the whole all data are presented in the main manuscript or additional supporting files.
