# Supplementary material for: The magnetic field strength and the force distance dependency of the magnetically controlled growing rods used for early onset scoliosis
Source: Sci Rep. 2023 Feb 21;13:3045. doi: 10.1038/s41598-023-30232-8 (PMC9944223; doi:10.1038/s41598-023-30232-8)
Supplement: Supplementary file 1 — Supplementary Figures. [file 41598_2023_30232_MOESM1_ESM.docx]

**Supplementary Information**

**The magnetic field strength and the force distance dependency of the**

**magnetically controlled growing rods used for early onset scoliosis**

Lars Diekhöner^1^ M.Sc. PhD, Charlotte Sommer Meyer^2,3^ MD, Søren Eiskjær^2,3*^ MD

^1^Aalborg University, Physics Group, Department of Materials and Production, Skjernvej 4A, 9220 Aalborg Ø, Denmark.

^2^Department of Orthopedic Surgery, Aalborg University Hospital, Hobrovej 18-22, 9000 Aalborg C, Denmark.

^3^Department of Clinical Medicine, Faculty of Medicine, Søndre Skovvej 15, 9000 Aalborg C, Denmark.

*Corresponding author: Søren Eiskjær, Kridtsløjfen 23, 4. 3, 9000 Aalborg C, Denmark. Phone: +4525520448. e-mail: spe@rn.dk

**Figure S1.** Force measured during actuation as a function of distance between ERC and MGCR-surface. The labels C-I refer to the 6 individual measurement. Also the average force (in black) is plotted (the same as given in Figure 3a of the main article). Data from wo different rods are shown: Rod 1 is a so-called “standard rod” and Rod 2 is an “offset rod”.

**Figure S2.** Measured magnetic field strength as a function of distance for the two new rods.

**Figure S3.** Correlation plot of the obtained maximum force, measured in the laboratory after the rod has been explanted, and the total expansion during use.

**Figure S4.** Correlation plot of the obtained maximum force, measured in the laboratory after the rod has been explanted, and the total time the rod has been used in the patient.
